# Functional Electrical Stimulation: A Possible Strategy to Improve Muscle Function in Central Core Disease?

**DOI:** 10.3389/fneur.2019.00479

**Published:** 2019-05-29

**Authors:** Pierpaolo Iodice, Simona Boncompagni, Laura Pietrangelo, Lucia Galli, Enrico Pierantozzi, Daniela Rossi, Aurora Fusella, Massimo Caulo, Helmut Kern, Vincenzo Sorrentino, Feliciano Protasi

**Affiliations:** ^1^CeSI-Met–Center for Research on Ageing and Translational Medicine, University G. d'Annunzio, Chieti, Italy; ^2^CETAPS–EA3832, University of Rouen Normandy, Mont-Saint-Aignan, France; ^3^Department of Molecular and Developmental Medicine, University of Siena and Azienda Ospedaliera Universitaria Senese, Siena, Italy; ^4^DNICS, Department of Neuroscience, Imaging, and Clinical Sciences, University G. d'Annunzio, Chieti, Italy; ^5^Ludwig Boltzmann Institute of Electrical Stimulation and Physical Rehabilitation, Vienna, Austria; ^6^Department of Medicine and Aging Science, University G. d'Annunzio, Chieti, Italy

**Keywords:** activities of daily living, congenital myopathy, excitation-contraction coupling, physiotherapy, genetic screening, ryanodine receptor type-1, training

## Abstract

Central Core Disease (CCD) is a congenital myopathy characterized by presence of amorphous central areas (or *cores*) lacking glycolytic/oxidative enzymes and mitochondria in skeletal muscle fibers. Most CCD families are linked to mutations in ryanodine receptor type-1 (RYR1), the gene encoding for the sarcoplasmic reticulum (SR) Ca^2+^ release channel of skeletal muscle. As no treatments are available for CCD, currently management of patients is essentially based on a physiotherapic approaches. Functional electrical stimulation (FES) is a technique used to deliver low energy electrical impulses to artificially stimulate selected skeletal muscle groups. Here we tested the efficacy of FES in counteracting muscle loss and improve function in the lower extremities of a 55-year-old female patient which was diagnosed with CCD at the age of 44. Genetic screening of the RyR1 gene identified a missense mutation (c.7354C>T) in exon 46 resulting in an amino acid substitution (p.R2452W) and a duplication (c.12853_12864dup12) in exon 91. The patient was treated with FES for 26 months and subjected before, during, and after training to a series of functional and structural assessments: measurement of maximum isometric force of leg extensor muscles, magnetic resonance imaging, a complete set of functional tests to assess mobility in activities of daily living, and analysis of muscle biopsies by histology and electron microscopy. All results point to an improvement in muscle structure and function induced by FES suggesting that this approach could be considered as an additional supportive measure to maintain/improve muscle function (and possibly reduce muscle loss) in CCD patients.

## Introduction

Central core disease (CCD), first described in 1956 (1), is one of the most common human congenital myopathies characterized by hypotonia and proximal muscle weakness (2). The typical form of dominantly inherited CCD is usually associated with a moderate degree of disability and carries an overall favorable prognosis, although clinical variability is often observed even within the same family (3–5). Diagnosis of CCD is confirmed by examination of muscle biopsies showing amorphous central areas (or *cores*) lacking glycolytic/oxidative enzymes and mitochondria (6), and disorganization of contractile and sarcotubular systems (7). Orthopedic complications are frequent in CCD and comprise congenital dislocation of the hips (8), scoliosis (9), and foot deformities (10).

About 90% of CCD cases are linked to mutations in the RYR1 gene (11–13), encoding for a tetrameric protein of about 2,200 KDa that forms the sarcoplasmic reticulum (SR) Ca^2+^ release channel of skeletal muscle, i.e., the ryanodine receptor type-1 (RyR1). RyR1 is a key protein in excitation-contraction (EC) coupling, the mechanism that allows the transduction of the action potential into Ca^2+^ release from the SR (14, 15). Mutations in the RYR1 gene are also responsible of malignant hyperthermia susceptibility (MHS), a hypermetabolic response to commonly used halogenated/volatile anesthetics (16). Many patients with CCD test positive for MHS, and, hence, should be considered at risk during general anesthesia.

No treatment is currently available for CCD and management of patients is essentially supportive based on a multidisciplinary approach. Usually, orthopedic complications limit the ability of CCD adult patient to perform physical exercise (2). Regular physiotherapy aims to preserve muscle function and to prevent contractures, particularly those of the achilles tendon, which are common in CCD. Considering a tendency to exercise-induced myalgia in CCD, exercises involving a high-resistance load are not recommendable.

Results collected in mice carrying human mutations linked to CCD in humans have provided evidence that oxidative stress may represent a pathophysiological mechanism in CCD and other RYR1-related myopathies (17). Accordingly, the antioxidant N-acetylcysteine (NAC) has been proposed to represent a potential therapy for patients with RYR1 (17, 18). Based on these experimental evidences and considering that NAC has been approved by FDA as a drug for various indications, clinical trials have recently been started to test the efficacy of this treatment (19, 20).

An independent line of work has led us to consider Functional Electrical Stimulation (FES) technique as a possible treatment for CCD patients. FES is a technique that uses low energy electrical pulses to artificially generate body movements. FES was originally used to develop neuroprostheses that were implemented to permanently substitute impaired functions such as grasping, walking, bladder voiding, and standing in individuals with spinal cord injury (SCI), head injury, stroke and other neurological disorders (21–23). FES has been successfully used to rescue muscle structure and function in animal models (24–26) and to promote recovery of muscle mass, structure and function in the lower limb of SCI patients with complete lesion of the conus cauda (21, 27–29). Recently, we also considered the use of FES as a method to improve muscle function in elderly, in a study in which the effects of FES on muscle was analyzed at the functional, structural, and molecular level (30).

In the present study, we tested the efficacy of 26 months of FES training in counteracting muscle loss and improving structure and function in the lower extremities of a 55-year-old female patient diagnosed with CCD at the age of 44. Measurement of maximum isometric force (MIF) of leg extensor muscles, magnetic resonance imaging (MRI), a complete set of functional tests to assess mobility in activities of daily living (ADL), and finally the analysis of muscle biopsies by histology electron microscopy (EM) all point to an improvement of muscle mass, structure, and function induced by FES.

## Materials and Methods

### Genetic Screening

**Figure 1:**
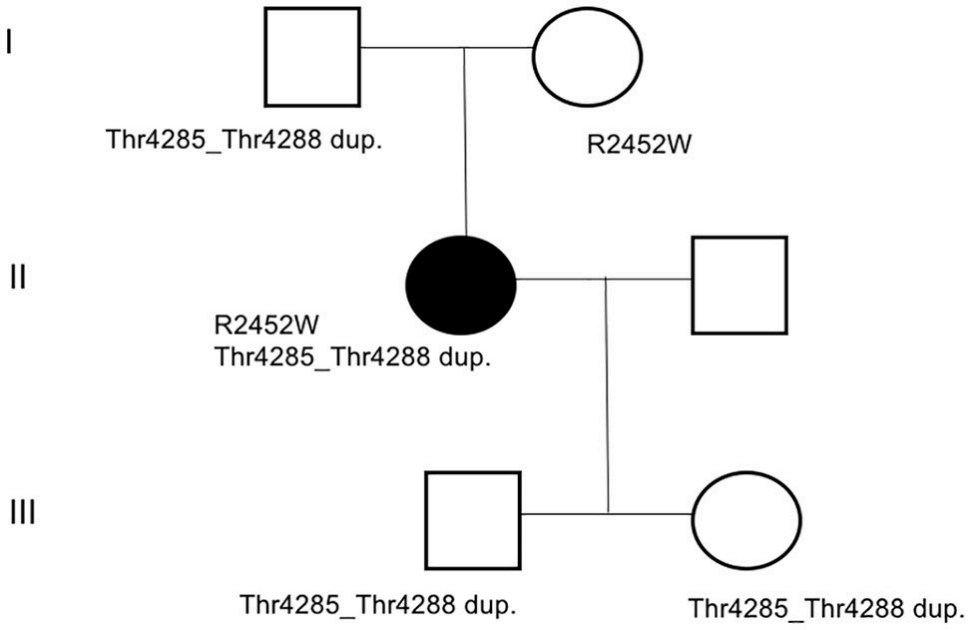
Family pedigrees. The black filled symbol represents the affected proband. Sequence analysis of the proband RYR1 gene identified a missense mutation (c.7354C>T) in exon 46, resulting in the substitution of an Arg in position 2452 with a Trp (p.R2452W), and a duplication c.12853_12864dupACGGCGGCCACG involving 4 amino acid residues (Thr, Ala, Ala, Thr, p.Thr4285_Thr4288dup) in exon 91. The genotype of family members tested is reported below each symbol.

Mutation screening by conventional Sanger sequencing on specific regions of the RYR1 gene was performed in all family members for whom DNA was available (Figure 1). In detail, primers were designed using Primer3 software (http://frodo.wi.mit.edu/primer3) to amplify all RYR1 exons (11). Genomic DNA was extracted from peripheral blood leucocytes by standard procedures (31, 32). Amplified DNA fragments were directly sequenced on an ABI3500 Genetic Analyzer (Applied Biosystems), using the Big-Dye Terminator v3.1 kit and analyzed with Sequencher 5.0 software (Gene Codes Corporation). DNA mutation numbering was based on cDNA reference sequence (NM_000540.2), taking nucleotide +1 as the A of the ATG translation initiation codon. The mutation nomenclature used follows that described at http://www.hgvs.org./mutnomen/.

### Functional Electrical Stimulation (FES) Training~Protocol

Stimulators and electrodes used in this study derives from devices developed in the EU project RISE (21, 28). The FES training procedure was developed by Dr. H. Kern and colleagues and previously applied on SCI patients with complete lesion of the conus cauda to promote recovery of muscle mass, structure and function in the lower limb (27–29, 33). The patient was provided with stimulators and electrodes at home, and after appropriate training and instructions, was able to perform stimulation at home. Two pairs of large electrodes, each having an area of 200 cm^2^, were strapped to the anterior surface of the thighs in proximal and distal positions (see Figure S2, for additional detail). Twitch contractions were elicited by biphasic rectangular current pulses lasting 100 to 150 ms and up to ±200 mA amplitude, representing an impulse energy up to ~2 Joules, to recruit fibers throughout the *Quadriceps Femoris* muscles. The long duration of the impulses needed for stimulation precluded the use of frequencies that would elicit tetanic contractions: training was initiated at 2 Hz, delivered for 15 min/day (series of 4 s on, 2 s off), 3 days/week. During the first/second month, muscle excitability progressively increased allowing an increase of 2 Hz stimulation, 2 × 10 min stimulation (2 min rest). As training proceeded, excitability of the muscle continued to increase, and from the third month onwards, training time stimulation were increased to 3 × 10 min (2 min rest).

### Experimental Design and Assessment of Effects of FES

**Figure 2:**
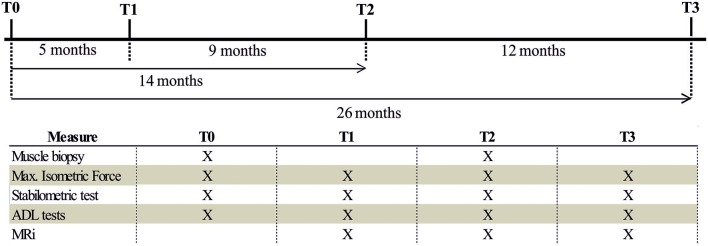
Flowchart of measurement procedure. Flowchart of the measurements performed in each period of the study (T0, T1, T2, and T3).

Muscle structure and ultra-structure was assessed by Magnetic Resonance Imaging (MRI), histology, and Electron Microscopy (EM). Muscle function was assessed by measuring maximum isometric force (MIF) and stabilometric test (ST), and using a complete set of functional tests to assess mobility in activities of daily living (ADL): Timed Up and Go Test (TUGT), 10 m Self Paced Walk Test (SPWT), and Short Physical Performance Battery (SPPB) as in Kern et al. (30). The effect of FES on muscle structure and function was assessed by doing all the tests mentioned above before the beginning of the 26 months of FES stimulation (T0), and at 3 different time points: T1, 5 months; T2, 14 months; T3, 26 months. See Figure 2 for additional detail.

#### Magnetic Resonance Imaging (MRI)

**Figure 3:**
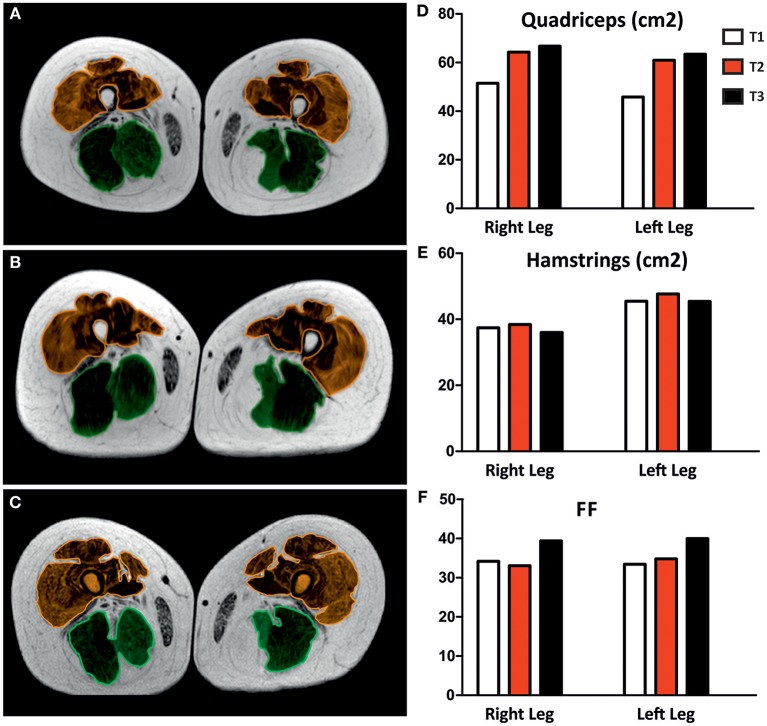
Magnetic Resonance Images (MTI) of thigh muscles. MRI was performed after 5 (**A**; T1), 14 (**B**; T2), and 26 months (**C**; T3) of home-based FES. The cross-sectional area of quadriceps (brown) and hamstrings (green) muscles was estimated and plotted in **(D,E)**, respectively. Histogram in **(F)** show the variation of fat fraction (FF) during FES treatment.

Magnetic Resonance Imaging was performed using a Philips Achieva 1.5 Tesla scanner (Best, The Netherland) using a quadrature body coil. The patient was comfortably placed in the supine position. Three axial TSE T2- and SE T1-weighted images (slice thickness 5 mm) were acquired at 3 different muscular levels: pelvic, thigh and leg. MRI was performed at 3 different time-points (T1, T2, and T3) after starting the FES training (see Figure 2). The cross-sectional area of quadriceps and hamstrings muscles (Figure 3) was estimated using Vue PACS software from Carestream Health, Inc. (Rochester, NY USA).

MRI was performed with a 1.5 Tesla scanner using a surface coil. Spin-echo (SE) T1 and Turbo Spin-echo (TSE) T2-weighted axial images (25 slices; slice thickness 10 mm; gap 10 mm) were conducted perpendicular to the long axis of the femur covering the entire leg from the hip to the knee joint. SE and TSE sequences were used because of the intrinsic different signal intensity between fat (hyperintense) and muscle (intermediate) in both T1 and T2 weighting. The duration time of the image acquisition was about 9 min: 1′ to obtain reference images; 3′13″ for SE T1 and 3′30″ for TSE T2 images. Axial images were then transferred to a workstation and 2 distinct regions of interest (ROI) were manually drawn by a radiologist with 5 years of experience in muscolo-skeletal disease to contour muscle and fat tissue. ROIs were drawn comparing T1 and T2-weighted images obtained at 3 different levels: proximal, intermediate and distal quadriceps. The area of the ROIs was considered to calculate the fat fraction in each level using the formula: Fat tissue ROIs/Total muscle ROIs.

#### Measurements of Maximum Isometric Force (MIF)

**Figure 4:**
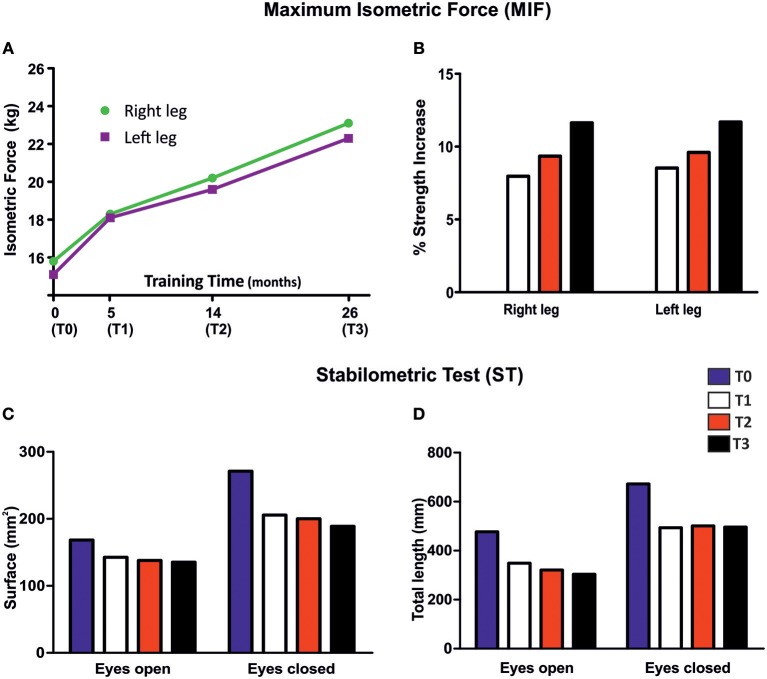
Maximum Isometric Force (MRI) and Stabilometric Test (ST). **(A)** Measurements of MIF based on leg extension movement before (T0) and after 5 (T1), 14 (T2), and 26 (T3) months of FES training. **(B)** Variations in MIF expressed as percentage, where values from T0 were taken as 0%. **(C,D)** Effect of FES treatment on surface (mm^2^) and total length (mm) in ST (52.2 s), in eyes open and eyes closed configuration, before (T0), and after 5 (T1), 14 (T2), and 26 (T3) months of home-based FES training.

Isometric measurements of force produced during leg extension was assessed using a leg extensor machine (Teca srl; Caldari Stazione CH, Italy) equipped with a load cell (Globus Italia; Codognè TV, Italy). Strength of the left and right knee extensors was measured at T0, T1, T2, and T3 of the study (Figure 4A). Briefly, the subject was positioned with hip at 90° flexion, the knee at 60° flexion (full knee extension = 0) and the arms crossed at the chest. The shank brace was positioned on the distal 1/3 of the lower leg and the trunk fixed with a seat belt tightening system. The subject was instructed to push alternating with one leg as fast and as hard as possible against a shank support separated through 1-min rest. In each case the maximal voluntary contraction was sustained for 3 s. All measurements were repeated three times for the right and left leg. The best value of each leg was taken for statistical analyses (34).

#### Stabilometric Test (ST)

Stability was evaluated using a static force platform (Globus Italia; Codognè TV, Italy) at T0, T1, T2, and T3 of the study (Figure 4B). During the assessment of static stability subject stood barefoot in a natural position, arms at their sides and forefoot open to 30° with eyes open facing a target placed 1.5 m away and eyes closed. The ground reaction force vectors were analyzed to calculate the center of pressure (COP) position (35). To calculate mean value of the excursions of COP, 3 trials were performed for each condition, each lasting 51.2 s, in accordance with the guidelines of the French Posturology Association. The sampling frequency was 120 Hz. The key dependent variables of the COP displacement are: its surface area and its total length, taken as the surface of the confidence ellipse enclosing 90% of COP sway (36).

#### Activities of Daily Living (ADL) Tests

**Figure 5:**
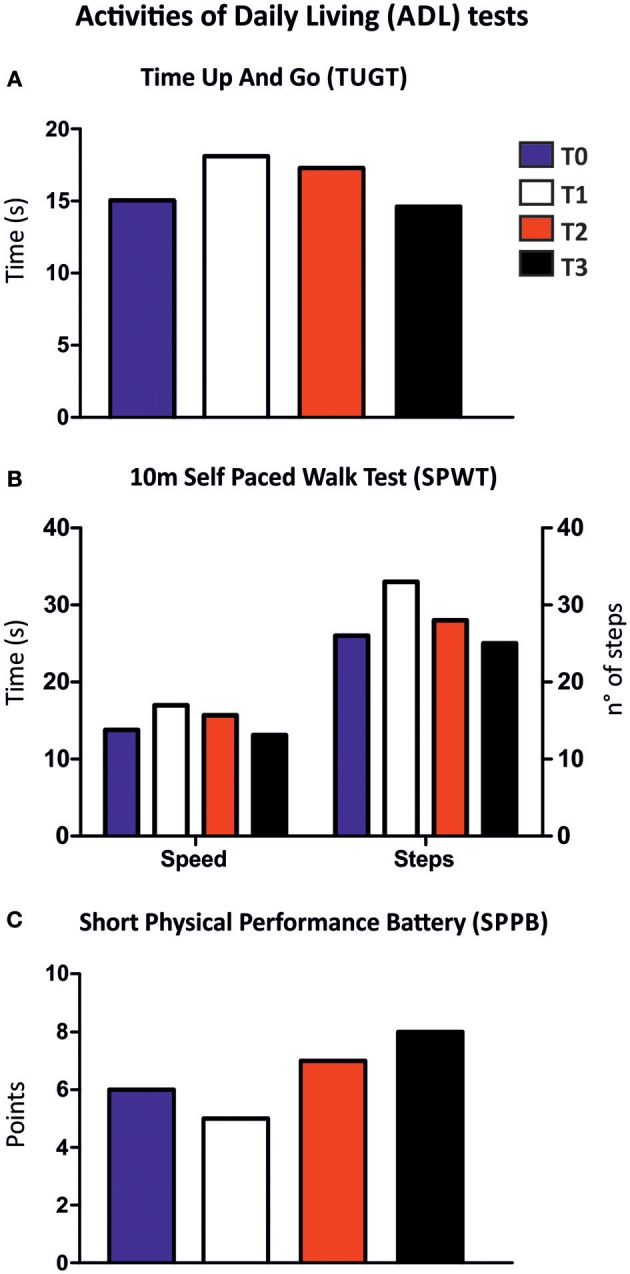
Activities of daily living (ADL) tests. Results of ADL tests of before (T0) and after 5 (T1), 14 (T2), and 26 (T3) months of home-based FES training: **(A)** 10 m self-paced walk test; **(B)** TUGT, time up and go test; and **(C)** SPPB, short physical performance battery.

A complete set of tests to access mobility in ADL was performed as in Kern et al. (30) to evaluate the effect of FES at T0, T1, T2, and T3 of the study (Figure 5):

1. *Timed-Up-And-Go-Test (TUGT)*. The subject was asked to stand up from a standard chair, walk a distance of 3 m, turn around, walk back to the chair and sit down again as fast as possible. The patient used her usual footwear and no assistive walking device. Timing, using a stop watch Track Pro (Conrad Electronics; Hirschau, Germany), begins when the subject starts to leave the chair back and ends when she was seated on the chair again (37).
2. *10 m Self Paced Walk Test (SPWT)*. The patient was asked to walk a distance of 10 m with either: (i) her habitual speed (i.e., the self-chosen normal speed of walking); or (ii) at her fastest walking speed. Each task was performed three times. Parameters of gait (average walking speed, average step length, and average step cadence) for each 10 m walking trial were calculated using a combination of a standard stop watch Track Pro (Conrad Electronics; Hirschau, Germany), counting the number of steps and subjective estimation of the remained part of the last step as described. The mean values of the parameters of three trials of each speed were taken for further analyses (30).
3. *Short-Physical-Performance-Battery (SPPB)*. The SPPB used for this study evaluates the lower extremities function by using tests of gait speed (the fastest time of two 4 m walk), standing balance (side-by-side, semi-tandem, and tandem stance for 10 s) and the time needed to rise from a chair for five times as quickest as possible with the arms folded across their chest. In each test the subject can reach a maximum score of 4. The sum of the three components comprised the final SPPB score with a possible range from 0 to 12 points (38).

#### Muscle Biopsies

Needle muscle biopsies were harvested through a small skin incision taken from the right *Vastus Lateralis* (VL) muscle of the patient at T0 and at T2 of the study. Biopsies were taken using a semi-automatic needle Precisa 13 Gauge (Hospital Service; Rome, Italy) as previously described by Franzini-Armstrong and collaborators (39). All the resulting specimens were then fixed for either light or Electron Microscopy (EM).

#### Histological Analysis and Electron Microscopy (EM)

Muscle biopsies were kept for ~3 min at room temperature in a “K acetate” solution (150 mM K acetate, 5 mM MgSO_4_, 10 mM EGTA, 10 mM phosphate buffer, pH 7.2) as in Pietrangelo et al. (39) and were fixed in freshly prepared 3.5% glutaraldehyde in 0.1 M cacodylate (NaCaCO) buffer, pH 7.4. The fixed bundles of fibers were stored at 4°C, till embedding. Samples were rinsed in 0.1 M NaCaCO buffer and post-fixed for 1 h in a mixture containing 2% osmium tetroxide (OsO_4_) and 0.8% potassium ferrocyanide [K_3_Fe(CN)_6_]. Specimens were then rinsed with distilled water, rapidly dehydrated in graded ethanol and acetone, infiltrated with Epon 812-acetone (1:1) mixture, and then embedded in Epon 812 resin (40).

**Figure 6:**
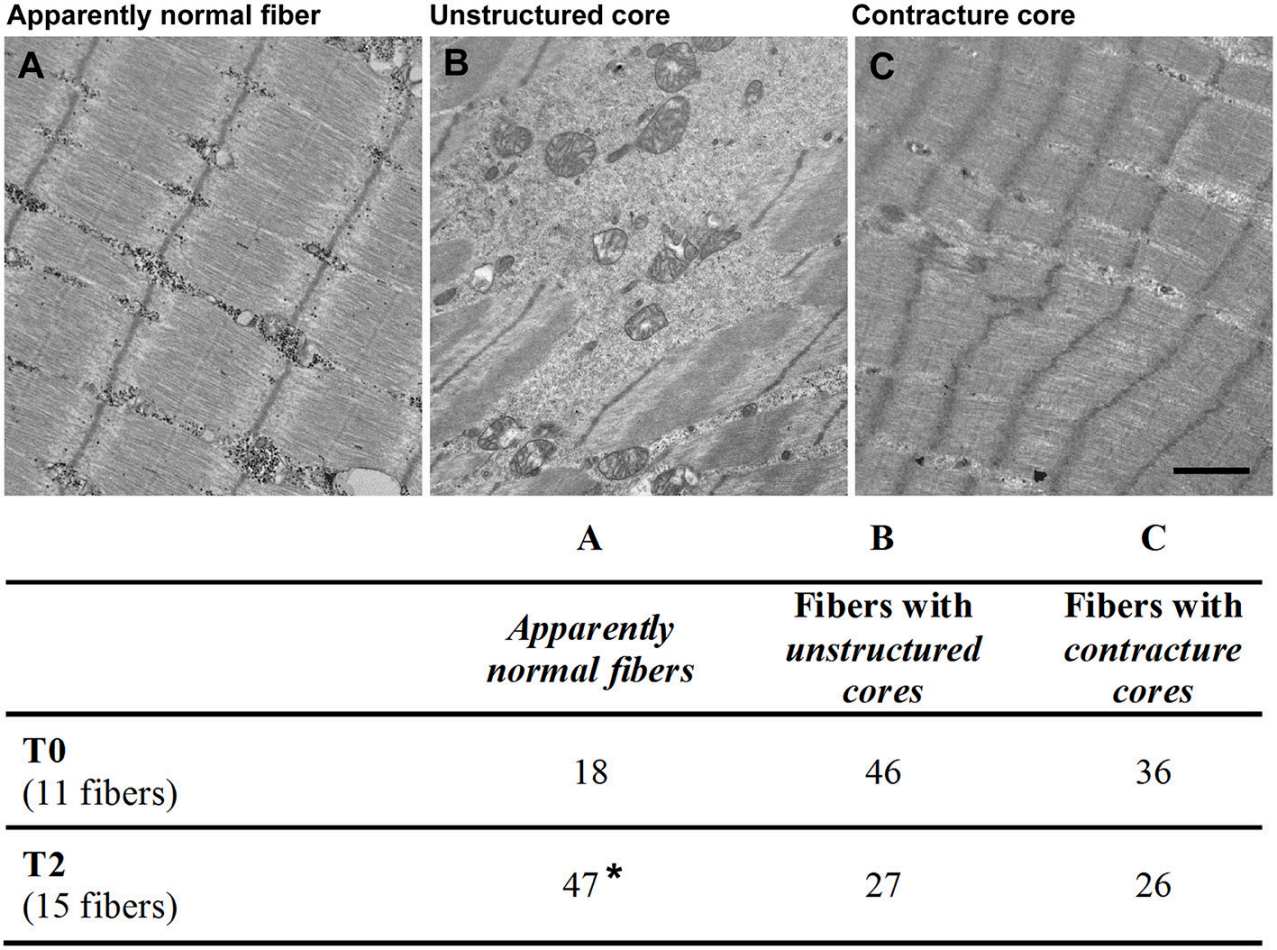
Appearance of skeletal fibers in electron microscopy (EM). Fibers were classified by EM for the quantitative analysis in different categories: **(A)** *apparently normal fibers* **(A)** with a typical pale-dark striation; **(B)** fibers with *unstructured cores*, presenting extended areas in which myofibrils were disrupted and replaced by amorphous cytoplasmic material; **(C)** fibers with *contracture cores*, with extended regions with laterally-packed and over-contracted myofibrils. Scale bar: 1 μm. Percentage, before (T0) and after 14 months (T2) were respectively reported in column A, B, and C. Data are expressed as % of total fibers analyzed; *p < 0.05 vs. T0.

For EM (Figure 6), ultrathin sections (50 nm) were cut with a Leica Ultracut R (Leica Microsystem; Vienna, Austria) using a Ultra Diatome diamond knife (Diatome Ltd.; Biel, Switzerland) and stained in uranyl acetate and lead citrate solutions. Sections were examined with a FP 505 Morgagni Series 268D electron microscope (FEI Company; Brno, Czech Republic) at 60 kV, equipped with a Megaview III digital camera and AnalySIS software Olympus Soft Imaging Solutions GmbH (Munster, Germany).

**Figure 7:**
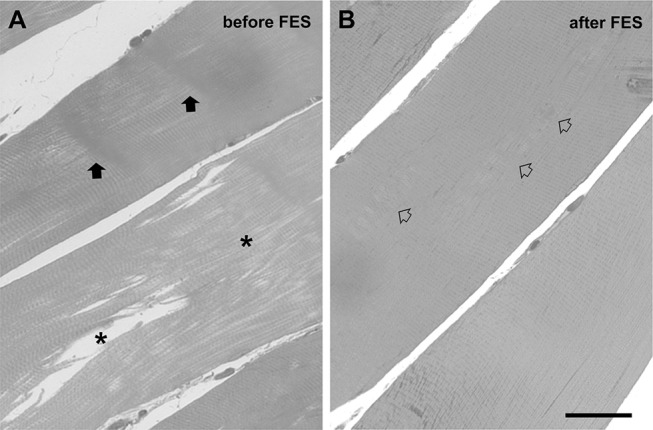
Histological images from biopsies before **(A)** and after FES training **(B)**. Stars and arrows in **(A)** mark respectively fibers with *unstructured cores* and fibers with *contracture cores*. Empty arrows in **(B)** points to regions in which striation is not yet completely rescued. Scale bar: 20 μm.

For histological analysis (Figure 7), semithin sections (700 nm) were cut with a Leica Ultracut R (Leica Microsystem; Vienna, Austria) using a Histo Diatome diamond knife (Diatome Ltd.; Biel, Switzerland) and stained with a 1% Toludine blue O, 1% Sodium Borate Tetra solution in distilled water for 3 min on a hot plate at 55–60°C. After washing and drying, sections were mounted with mounting medium DPX Mountant for Histology (Sigma-Aldrich; Milan, Italy) and observed with a Leica DMLB light microscope R (Leica Microsystem; Vienna, Austria) equipped with a Leica DFC 450C digital camera and Leica Application Suite v4.1.0 (Leica Microsystem; Vienna, Austria). Fibers were examined for the presence of either contracture or unstructured cores as in Boncompagni et al. (41).

## Results

### Case Presentation

The 55-year-old Caucasian woman enrolled in the study was diagnosed with CCD (CCD; OMIM# 117000) at the age of 44 (Center for Neuromuscular Diseases; Ospedale Clinicizzato SS. Annunziata, I-66100 Chieti, Italy). CCD was confirmed by histological examination of muscle biopsies at the time of the initial diagnosis (not shown). Presence of areas of internal disarray in skeletal muscle fibers was also confirmed in the present study (please see Figure S1, for more detail). The patient reported that her disease had been aggravated by orthopedic complications (scoliosis diagnosed at the age of 17 years) that limited her daily activities to a point in which she retired from working activities at the age of 54. At the time of enrolment in the present study a medical check-up was performed (anthropometric data, medical aptitude, and anamnesis): her vital signs were all within normal limits, except for a moderately high body mass index (Table 1). The score of muscle strength test by the Medical Research Council scale was 3/5 in both upper and lower limbs (42). Patient had a waddling gait and difficulty in climbing stairs.

**Table 1:** Anthropometric measurements.

	**T0**	**T1**	**T2**	**T3**
Weight	61.1	65.5	69.3	64.5
Height	145	145	145	145
BMI	29.01	31.15	32.96	30.71

*Data reported are the anthropometric measurements of patient during the study, before (T0) and after 5 (T1), 14 (T2), and 26 (T3) months of home-based FES training*.

### Genetic Screening

Sequence analysis of the RYR1 gene identified a missense mutation (c.7354C>T) in exon 46 resulting in the substitution of the Arg in position 2452 with a Trp (p.R2452W) and a duplication (c.12853_12864dup12, p.Thr4285_Thr4288dup) in exon 91, involving 4 amino acid residues (Thr, Ala, Ala, Thr, p.Thr4285_Thr4288dup) in exon 91 was also observed. To test how these two mutations segregate in the family, DNA samples from relatives were obtained and analyzed by direct sequencing (Figure 1). This analysis indicated that mutations are independently transmitted in the family and that the proband inherited the c.12853_12864dup12 duplication from the father and the c.7354C>T mutation from the mother. Analysis of the daughter and the son of the proband, indicated that both inherited only the duplication from the father. From a clinical point, neither the parents nor the sons of the proband presented signs of myopathy or CCD.

### FES Training Protocol

Stimulators and electrodes used in this study derives from devices developed in the EU project RISE (21, 28). The patient was provided with stimulators and electrodes, and after appropriate training and instructions, was able to perform FES at home (please see Figure S2, for more detail). The effect of FES on muscle function and structure was assessed by measurement of maximum isometric force (MIF) of leg extensor muscles, magnetic resonance imaging of lower extremities (MRI), a complete set of functional tests to assess mobility in activities of daily living (ADL) and, finally, histological and electron microscopy (EM) analysis of muscle biopsies. Functional tests and structural studies were performed before the beginning of the 26 weeks of FES stimulation (T0), and at 3 different time points during FES training: T1 (5 months), T2 (14 months), and T3 (26 months). See section Materials and Methods and Figure 2 for additional detail about stimulation protocols and adjustment of parameters during the different phases.

### Effects of FES Treatment on Thigh Muscles as Assessed by MRI

Figures 3A–C shows representative color MRI of thigh muscles after 5, 14, and 26 months of FES training (T1, T2, and T3, respectively). Unfortunately, data at T0 were not collected. Data collected from measurements of MRI scan images are plotted in Figures 3D–F. The average cross sectional area (CSA) of the quadriceps (brown in Figures 3A–C) increased of about 15% from T1 to T2 and of about 18% at T3 (Figure 3D). The size of quadriceps muscle increased similarly in the right and left leg, while no changes were detected in the hamstrings muscles, i.e., the posterior muscles of the thigh (green in Figures 3A–C). Note that, during stimulation, the surface electrodes were placed on the front part of the thigh, in correspondence to quadriceps, and, as a consequence, hamstrings were not directly stimulated by FES. Figure 3F shows increased percentage of free fat mass at T3. These results indicate that FES treatment increased muscle mass at T2 and T3 and reduced fat fraction area at T3. See Table S1 for additional detail.

### Functional Tests

To investigate the beneficial effect of FES treatment on muscle strength and balance of the patient, maximum isometric force (MIF) and orthostatic balance during a stabilometric test (ST) were tested.

#### Maximum Isometric Force (MIF)

Isometric strength of leg extensor muscles was measured before (T0), and after 5 (T1), 14 (T2), and 26 (T3) months of FES treatment (Figures 4A,B). MIF increased in both legs (Figure 4B), beginning at 5 months (T1, 8%) of treatment and continuing during the entire length of the study (9% at T2, and 11% at T3).

#### Stabilometric Test (ST)

Surface area and length of center of pressure (COP) measured before (T0) and after 5 (T1), 14 (T2), and 26 (T3) months of FES treatment are shown in Figures 4C,D. In general, COP displacement in both tested conditions (eyes open and eyes closed), revealed an important decrease in surface area (amplitude of oscillatory movements) and in total length (frequency of oscillatory movements) at T1 that persisted in the following periods (T2 and T3). These results indicate that the capability of the patient to maintain balance while standing was already improved after only 5 months of FES training.

### Activities of Daily Living (ADL) Tests

We monitored whether improved muscle force and balance while standing (Figure 4) was associated with an effective improvement of the patient's Activities of Daily Living (ADL). A complete set of tests to assess mobility of the patient was employed as in Kern et al. (30) to evaluate the effect of FES at the different time points of the study: Timed-Up-And-Go-Test (TUGT), 10 m Self Paced Walk Test (SPWT), and Short Physical Performance Battery (SPPB) (Figure 5). The results of these tests were slightly contradictory, as at T1 and T2 the results collected point to a worsening of the patient's performance, while a possible improvement was detected at T3: (a) shorter total times in TUGT test (−3.2%; Figure 5A); (b) an improvement in 10 m walking performance, time needed to cover 10 m (speed) and number of steps during SPWT decreased (−5.1% and −4.2%, respectively; Figure 5B); (c) higher score obtained in the SPPB (6 vs. 8 points; Figure 5C). Taken together, these data suggest a slight improvement of the patient's ADL at T3. The reason for the partially negative outcome at T1 and T2 will be further discussed below (see section Discussion).

### Histological and EM Analyses of FES Treatment on Thigh Muscles

Muscle biopsies of *Vastus Lateralis* (VL) muscle were analyzed by EM (Figure 6) and by histology for quantitative analysis (Figures 6, 7; an image in cross section at lower magnification is provided in Figure S1) before (T0) and after 14 months of FES training (T2).

At the EM analysis (Figure 6) fibers presented with different appearances: (a) *apparently normal fibers*, which displayed a normal cross striation pattern of the skeletal muscle fibers (Figure 6A); (b) fibers with *unstructured cores* (Figure 6B), extended areas in which myofibrils were disrupted and replaced by amorphous cytoplasmic material; and (c) fibers with *contracture cores* (Figure 6C), regions with laterally-packed and/or over-contracted myofibrils. In *unstructured cores*, mitochondria were often damaged (Figure 6B), while in *contracture cores* both mitochondria and SR were scarce or completely missing (Figure 6C). Figure 7 shows two representative histological images from biopsies before (Figure 7A) and after the 14 months of FES training (Figure 7B): we used this approach to evaluate whether FES improved fiber structure and/or reduced the number of cores. Before FES training (T0), only 18% of skeletal fibers analyzed displayed the normal cross striation pattern, with the remaining 82% presenting structural abnormalities, i.e., either *unstructured* or *contracture cores* (see Figure 6). Following FES training, while recovery was definitely not complete, the percentage of fibers containing cores was significantly reduced from 82 to 53% (Figure 6, columns B and C), accompanied by a parallel increase (from 18 to 47%) in the percentage of fibers with a fairly normal appearance (Figure 6, column A).

## Discussion

### Background

CCD is a disorder usually associated with a mild to moderate degree of disability. Weakness is usually slowly progressive, but orthopedic complications often limit the ability of CCD adult patients to perform physical exercise (2). While the use of anti-oxidants is being considered for the treatment of patients (17, 18, 20), CCD has currently no cure, and the development of strategies aiming to maintain muscle mass and strength could be of help. FES has been previously used to rescue or maintain muscle functions in individuals with no, or limited, capability to perform physical exercise: FES was indeed beneficial to improve muscle function in patients with spinal cord injury (27–29) and in elderly individuals (30).

### Main Findings of the Study

Here we tested the possibility of using FES to improve muscle function in a 55-year-old female patient diagnosed with CCD at the age of 44 by providing stimulators and electrodes to the patient at home (see Figure S2). Sequence analysis of the RYR1 gene in the patient identified a missense mutation (c.7354C>T) in exon 46, which had been previously reported in an individual with MHS (43) and a duplication (c.12853_12864dup12) in exon 91 (Figure 1). This duplication has not been reported in the literature, but occurs in a region of the RYR1 gene where other duplications associated with MHS have been reported (44, 45). Actually, the duplication reported here partly overlaps with the one we report in Levano et al. (45). The patients inherited the c.12853_12864dup12 from the father, while the c.7354C>T mutations was inherited from the mother. Absence of clinical evidence of myopathy in the family members carrying only one of the two mutation in the RYR1 gene and evidence of central core in the proband carrying two mutations agrees with a recessive form of CCD (19, 20, 46, 47).

The results of tests and experiments aiming to evaluate the effects of FES on muscle function and structure were reasonably encouraging. According to MRI analysis, FES treatment resulted in a selective increase in muscle mass (at T2 and T3) in stimulated quadriceps (Figure 3), while no improvement was exerted on the hamstring due to an unfavorable position of the electrodes used to stimulate tight muscles. A reduction in fat mass area was observed at T3. In addition, functional tests reported in Figure 4 also led to some positive outcomes:

- Maximum isometric force (MIF) of leg extensors improved in both legs, already significantly at the fifth month of stimulation (8% at T1), and continued to increase steadily until the end of the treatment to achieve an improvement of 11% at 26 months (T3) (Figure 4). The increase in force may result from the combined effect of an increase in muscle mass (Figure 3; discussed above) and of the improvement in ultrastructure of skeletal fibers (Figures 6, 7). The qualitative analysis was supported also by quantitative data revealing a significant decrease in the percentage of fibers presenting structural cores from T0 to T2: 82 vs. 53% (Figure 6).
- Stabilometric test (ST) also showed an improvement in balance of the patient in both eyes-open and eyes-closed conditions (Figure 4). Worth to notice also that already at T1 (5 months) FES induced improvement in the ST test. These results suggest that the subject's balance may depend on muscle mass and strength (Figures 3, 4), as previously suggested (48, 49). The comparison of results in eyes-open and eyes-closed conditions (a) indicates that muscle force (likely needed to maintain balance) has a relatively greater role in eyes-closed condition, (i.e., the condition without visual perceptual information) and (b) suggests that the strength of lower limbs is crucial for the transfer of balance control skills to all environmental situations (50).

The results of the battery of ADL tests were less straightforward than the ones collected with other approaches, as at T1 and T2 there were no evidence of improvement, but rather a general worsening of performance in the various tasks, while at T3 scores were improved compared to T0 (Figure 5). The reason for this partial negative outcome is unclear. However, one of the possible explanations is the low back-pain lamented by the patient for several months during the trial, a problem that had temporally caused a significant reduction in her daily walking activities. However, it is important to underline that despite the low back pain, at the end of the FES treatment (T3) all ADL tests showed a slight improvement in the patient's performance. The result of ADL tests (Figure 5) are considered in literature as a parameter to score the degree of independence (27, 51, 52) and an indirect index of muscle strength (52).

## Conclusions

The case report presented in this study represents an attempt to find possible strategies for CCD patients to exercise muscles in the lower extremities. Our results confirmed previous finding on the effectiveness of the FES treatment in increasing muscle strength in pathological subjects (27). The treatment proved to have a strong compliance by the patient, which make it to be a favorite choice for long-term home care treatments. Results of this study are moderately encouraging and suggest that FES could be considered as an additional supportive measure to maintain/improve muscle function in CCD patients.

## Ethics Statement

The patient in this study has provided written informed consent for both participations in the study and the use of data and material for publication. The study was approved by G. d'Annunzio University of Chieti ethics boards (EK08-102-0608).

## Author Contributions

FP conceived and directed the study. PI managed FES training and performed all functional measurement and tests of ADL. SB, LP, and AF performed histological and EM analysis. EP, LG, DR, and VS performed the genetic screening of patient and family members. MC performed MRI. HK provided the electrical stimulation devices and designed the training protocols. Finally, PI, FP, SB, DR, and VS wrote the manuscript.

### Conflict of Interest Statement

The authors declare that the research was conducted in the absence of any commercial or financial relationships that could be construed as a potential conflict of interest.

## Abbreviations

ADL: activities of daily living
CCD: central core diseases
EM: electron microscopy
FES: functional electrical stimulation
MIF: maximum isometric force
MRI: magnetic resonance imaging
RYR1: ryanodine receptor type-1
SPPB: short physical performance battery
SPWT: self-paced walk test
ST: stabilometric test
TUGT: timed up and go test.
