# Supplementary material for: Functional Electrical Stimulation: A Possible Strategy to Improve Muscle Function in Central Core Disease?
Source: Front Neurol. 2019 May 29;10:479. doi: 10.3389/fneur.2019.00479 (PMC6548841; doi:10.3389/fneur.2019.00479)
Supplement: Supplementary file 1 [file Image_1.pdf]

Supplementary Material

Figure S1: ***Histological images in cross section of muscle biopsy before (T0) beginning of FES training.***

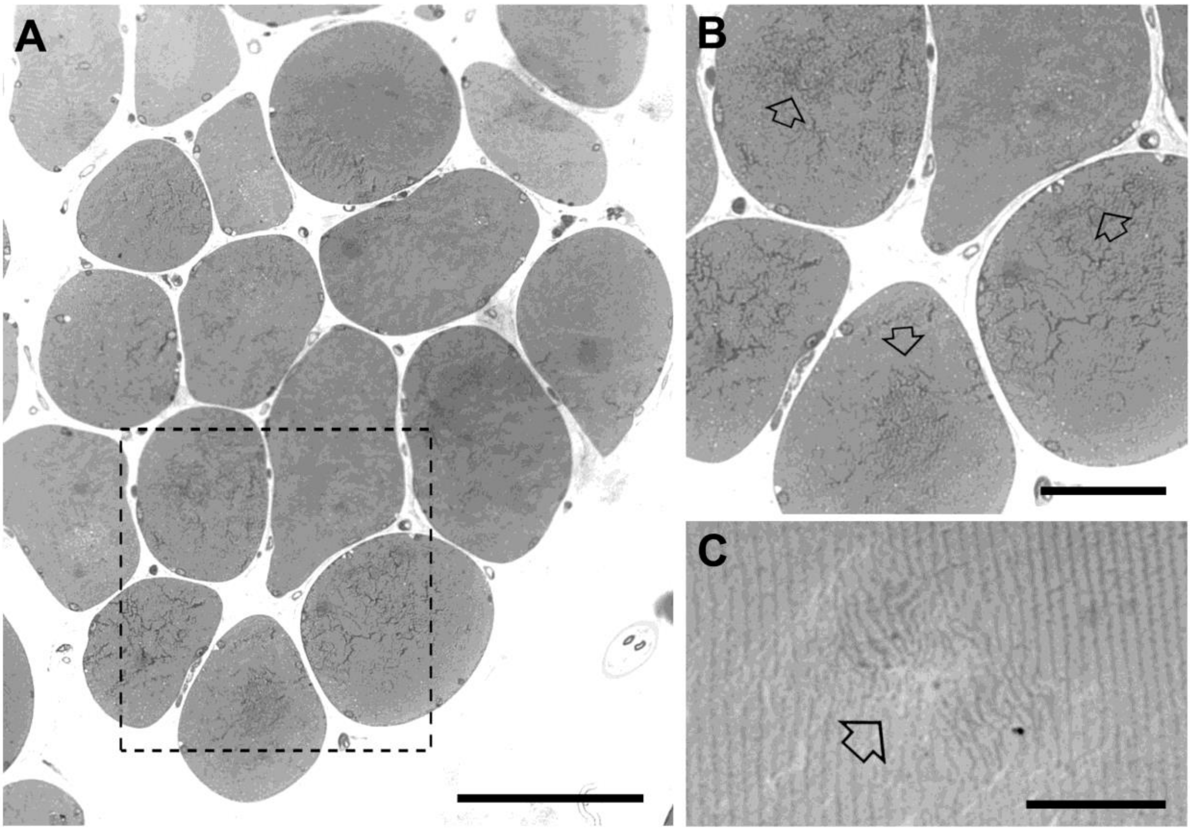

Arrows in point to internal fiber areas in which disarray of the contractile elements is more evident. Scale bars: A, 100 µm; B, 40 µm; C, 20µm.
