# Supplementary material for: Functional Electrical Stimulation: A Possible Strategy to Improve Muscle Function in Central Core Disease?
Source: Front Neurol. 2019 May 29;10:479. doi: 10.3389/fneur.2019.00479 (PMC6548841; doi:10.3389/fneur.2019.00479)
Supplement: Supplementary file 2 [file Image_2.pdf]

Supplementary Materials

- Figure S2: *Electrodes placement during FES training*.

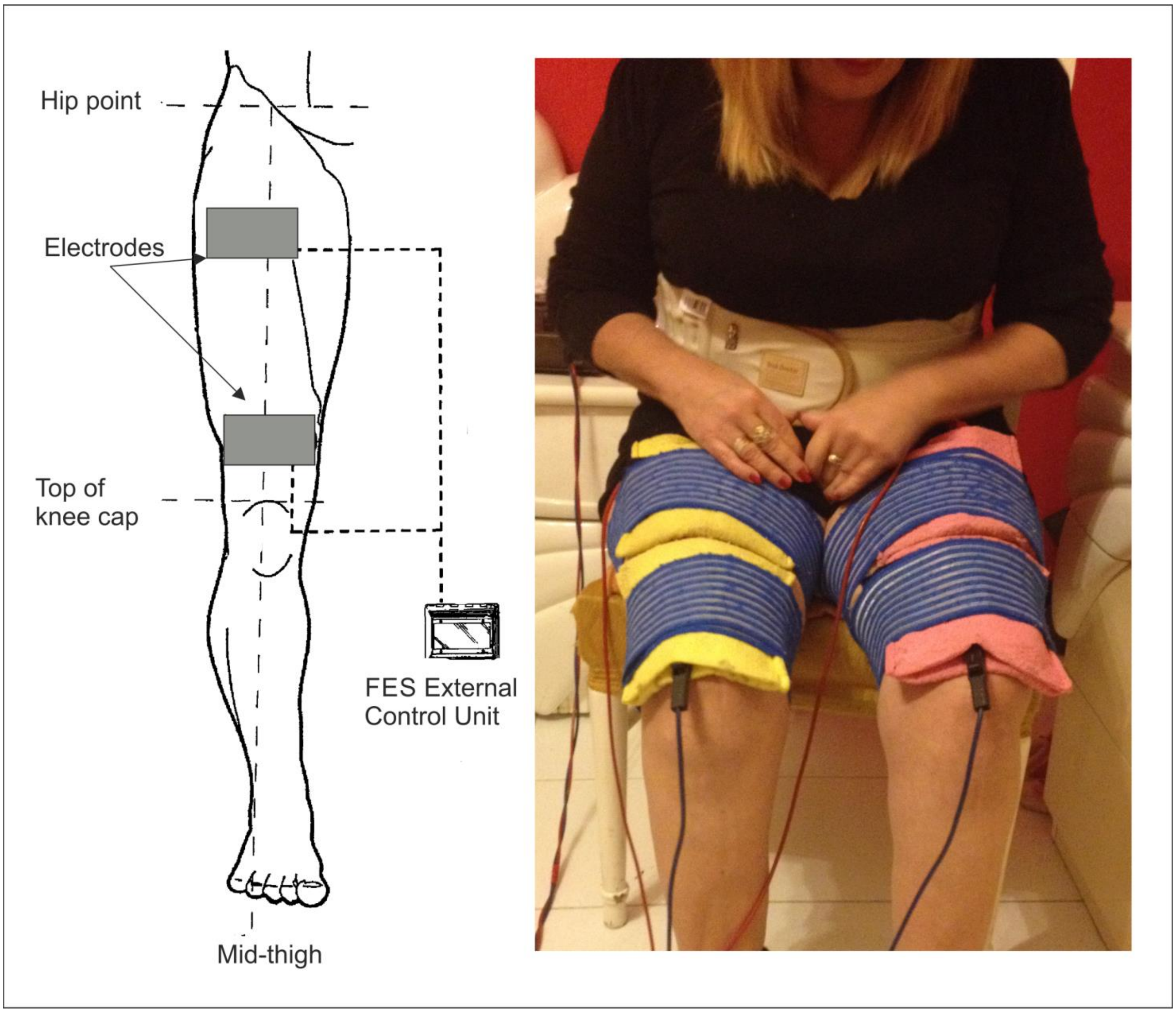

The patient was provided with stimulators and electrodes, and after appropriate training and instructions, was able to perform stimulation at home. Two pairs of large electrodes, each having an area of 200 cm^2, were strapped to the anterior surface of the thighs in proximal and distal positions.
