# Supplementary material for: Functional Electrical Stimulation: A Possible Strategy to Improve Muscle Function in Central Core Disease?
Source: Front Neurol. 2019 May 29;10:479. doi: 10.3389/fneur.2019.00479 (PMC6548841; doi:10.3389/fneur.2019.00479)
Supplement: Supplementary file 3 [file Table_1.pdf]

Supplementary Materials

Table S1: *Cross-sectional area of quadriceps and hamstring assessed by MRI.*

	T1		T2		T3	
	Rx Leg	Lx Leg	Rx Leg	Lx Leg	Rx Leg	Lx Leg
Quadriceps (cm^2)	51.52	45.81	64.27	60.99	66.77	63.45
Hamstrings (cm^2)	37.41	45.49	38.44	47.67	36.01	45.42
Tot. Mass (cm^2)	260.18	273.16	310.45	312.27	260.93	272.46
Fat-Free (cm^2)	88.93	91.3	102.71	108.66	102.78	108.87
Fat-Fraction (cm^2)	171.25	181.86	207.74	203.61	158.15	163.59
% Fat-Free Mass	34.18	33.42	33.08	34.80	39.39	39.96

Cross-sectional area of thigh muscles analysis after 5 (panel A; T1), 14 (panel B; T2), and 26 months (panel C; T3) of home-based FES home-based. Total Mass, Fat-Free, and Fat-Fraction of right (Rx) and left (Lx) thigh muscles are reported for each time points.
